# Specialized Rehabilitation Programs for Children and Adolescents with Severe Disabling Chronic Pain: Indications, Treatment and Outcomes

**DOI:** 10.3390/children3040033

**Published:** 2016-11-21

**Authors:** Lorin Stahlschmidt, Boris Zernikow, Julia Wager

**Affiliations:** 1 German Paediatric Pain Centre, Children’s and Adolescents’ Hospital, 45711 Datteln, Germany; 2 Department of Children’s Pain Therapy and Paediatric Palliative Care, Faculty of Health, School of Medicine, Witten/Herdecke University, 58448 Witten, Germany; l.stahlschmidt@deutsches-kinderschmerzzentrum.de (L.S.); b.zernikow@kinderklinik-datteln.de (B.Z.)

**Keywords:** chronic pain, indication, rehabilitation programs, specialized pain treatment, pediatric, effectiveness

## Abstract

Children and adolescents with highly disabling chronic pain of high intensity and frequency are admitted to specialized pain rehabilitation programs. Some barriers to obtaining this specialized care include a lack of availability of treatment centers, a perceived social stigma and individual barriers such as socioeconomic status. Specialized rehabilitation programs for severe disabling chronic pain worldwide have similarities regarding admission criteria, structure and therapeutic orientation. They differ, however, regarding their exclusion criteria and program descriptions. The short- and long-term effectiveness of some rehabilitation programs is well documented. All countries should promote the establishment of future pediatric pain centers to improve the health care of children and adolescents suffering from severe chronic pain. Standardized reporting guidelines should be developed to describe treatments and outcomes to enable comparability across treatment centers.

## 1. Introduction

Approximately 3% to 5% of children and adolescents experience strong negative consequences of chronic pain [1]. They miss a significant amount of school and experience a decline in their grades, a considerable reduction in their quality of life and psychological distress [1,2,3,4,5]. The long-term prognosis of these children and adolescents is poor. Without effective treatment, their chronic pain is likely to continue into adulthood [6,7,8]. This process of chronification can potentially be interrupted with specialized pain treatments. However, not all children and adolescents seek treatment when they suffer recurrent pain [9,10]. One important and very robust predictor of health care utilization is high pain-related disability [1,10,11,12]. Additional factors associated with health care utilization include pain characteristics, such as high pain frequency or high pain intensity [1,10,11,12]. 

In many health care systems, the first point of contact is typically primary care, with subsequent referrals to secondary or tertiary care [13,14]. However, some health care systems, e.g., those in the USA, do not follow this structured approach; in these types of settings, patients may self-refer and enter the system at any level [13]. Some of the children and adolescents who visit a primary care physician for recurrent or constant functional pain are referred to specialized pain treatment centers, while many patients are (mistakenly) referred to other specialists such as rheumatologists, neurologists, gastroenterologists or orthopedic surgeons [11,15,16,17]. Before presenting to specialized pediatric pain treatment centers, patients undergo a substantial number of medical visits and pain-related hospital stays [2,11,17,18]. Furthermore, other barriers to accessing specialized pain care exist, such as availability of care [19,20,21] and socioeconomic status [19,20]. A lack of understanding in society and among friends, family or even primary care physicians due to the invisible nature of pain may also present a barrier to specialized care [22]. In addition, the perceived stigma associated with psychological therapy can prevent patients from seeking specialized care with a psychological focus [22,23], especially if the patients believe that their problem is a physical one [23]. Pain location can represent a barrier to accessing specialized care as well, as not all pediatric pain programs treat all types of pain. 

**Figure 1:**
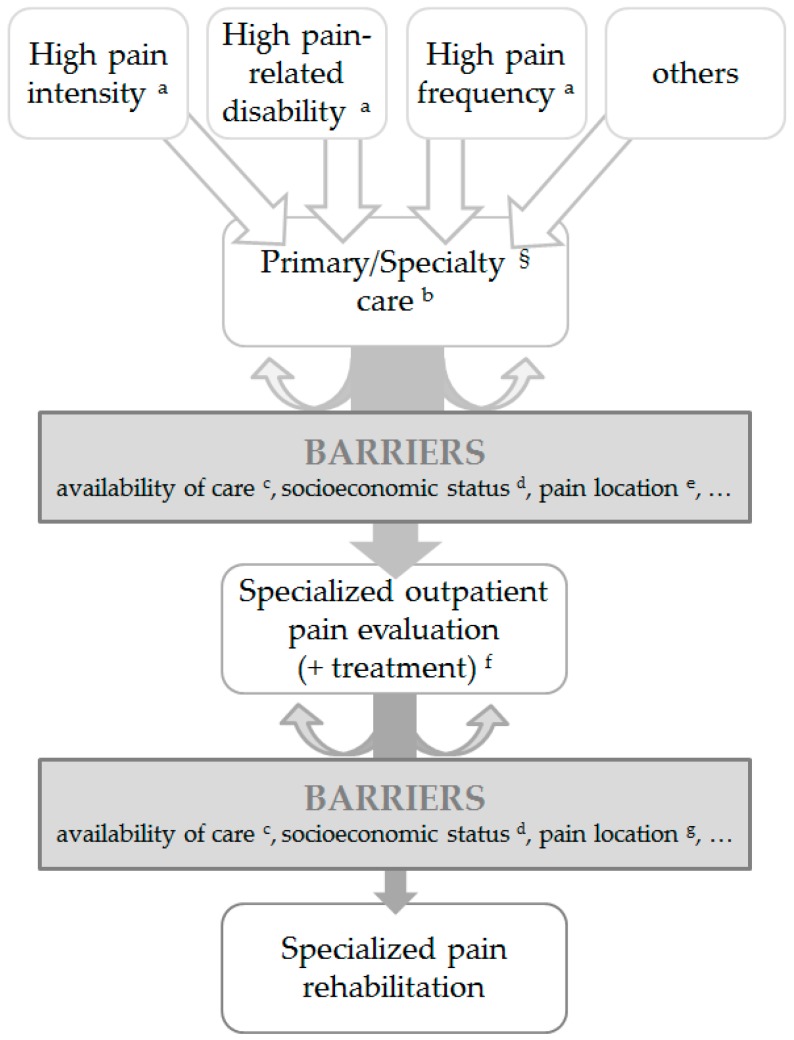
Usual course of health care utilization due to chronic pain. ^§^ e.g., rheumatologists, neurologists, gastroenterologists, orthopedic surgeon. ^a^ e.g., [1,10,11,12]; ^b^ e.g., [11,15,16,17]; ^c^ e.g., [19,20,21]; ^d^ e.g., [19,20]; ^e^ e.g., [26]; ^f^ e.g., [25,26,27]; ^g^ e.g., [28].

Specialized pain care provides treatment options with different levels of intensity. For patients with a moderate level of functional and emotional impairment, outpatient chronic pain treatment with limited intensity may be sufficient [24,25,26,27]. For severely disabled children and adolescents with chronic pain, an intensive specialized rehabilitation program is indicated. Figure 1 displays the usual course of health care utilization due to chronic pain. 

Currently, there are specialized pediatric pain centers in many countries around the world. According to the International Association for the Study of Pain [29], specialized pain clinics are characterized by the concurrent efforts of an interdisciplinary clinical team that is able to treat any type of pain disorder. The patient-centered treatment provided in these clinics is based on the best available evidence, and these clinics implement quality improvement efforts, for example, by routinely monitoring patient characteristics and outcomes (see [30] for an example of a successful implementation of such methods). Specialized pain centers also engage in research and academic teaching. According to the bio-psycho-social model of chronic pain, chronic pain treatment should always include medical, psychological and social treatment methods. There are pain clinics that specialize in only one pain disorder but fulfill all of the other criteria defined by the IASP. The American Pain Society additionally defined parent inclusion and school reintegration as particular requirements of specialized pediatric pain treatment [31]. The overall goal of pediatric chronic pain treatment is to assume an active self-management approach in coping with pain [32,33] and to enable age-appropriate daily activities despite pain [33,34,35].

In this review, we focus on specialized pain rehabilitation programs in particular and do not address specialized outpatient treatment. A recent systematic review showed strong positive results regarding the short-term effectiveness of specialized pain rehabilitation programs [36]. Here, we aim to provide a comprehensive overview of existing pain rehabilitation programs, their structure and their short- and long-term effectiveness. First, we present the criteria that suggest that intensive pain treatment is indicated. Second, we take an international perspective on the structure and the components of different pain rehabilitation programs worldwide to note their differences and similarities. Third, we provide a summary of the results regarding short- and long-term outcomes of pain rehabilitation programs. With the results presented in this review, we aim to promote the development of further specialized pain programs, to place single programs within a larger context and to advance global networking.

## 2. Method

We conducted a literature search based on a recent systematic review of intensive interdisciplinary pain treatment [36] initiated by our research group. This published systematic review integrated results from ten studies and had a strong focus on the outcomes of chronic pain treatment. In this review, we concentrated more on providing a comprehensive description of existing specialized rehabilitation programs and therefore further included referenced studies that were excluded from the systematic review, for example due to overlapping samples [25,37,38,39,40,41,42]. Additionally, we identified five more recent studies on this topic through a nonsystematic search in Medical Literature Analysis and Retrieval System Online (MEDLINE) and by conducting a manual search [16,28,34,43,44,45]. Overall, information about nine different rehabilitation programs from the USA, the UK, Australia and Germany was available. We extracted information from the included studies concerning the admission criteria of the rehabilitation programs and their structure, therapeutic orientation and treatment components. We further extracted information regarding outcomes according to the core outcome domains listed in the Pediatric Initiative on Methods, Measurement, and Pain Assessment in Clinical Trials (PedIMMPACT) recommendations [46].

## 3. Results

### 3.1. Indications for Specialized Rehabilitation Programs

The criteria for admission to specialized rehabilitation centers vary slightly between the rehabilitation programs worldwide. The inclusion criteria showed considerable similarities. Most programs agree that the most important criteria is for the pain to be persistent with a high intensity for at least three months [33,35,47] and for children and adolescents to be severely impaired by the pain in daily activities such as school attendance, sports or leisure activities [32,33,35,47,48,49]. As family environment may be an important factor in the development or maintenance of pain, patients’ and their parents’ motivation for and compliance with treatments are important requirements [35,47,48,49]. In addition, failure of prior outpatient treatment is often mentioned as a necessary admission criterion [32,47,49]. This factor is important for avoiding overtreatment. The exclusion criteria vary widely between the rehabilitation programs. Patients with specific psychiatric needs requiring further treatment are often excluded [32,35,49], as are patients requiring further medical assessments [33,35] and patients with a medical pathology/underlying disease [32,48] or a malignant disease [47,48]. If other effective medical options are available, this can also be an exclusion criteria [48]. Table 1 provides an overview of the criteria for the different rehabilitation programs introduced in the literature.

### 3.2. Treatment Components of Specialized Rehabilitation Programs

Specialized rehabilitation includes inpatient chronic pain treatment and intensive day-hospital approaches [36]. Most of these programs treat all types of pain disorders, although some specialize only in musculoskeletal pain [28,32]. Similar to the admission criteria described above, the rehabilitation programs vary slightly around the world; however, they agree on a number of core contents and structures. These programs mainly include operant and cognitive behavioral techniques, as well as some acceptance and commitment therapy-based approaches, and they consist of a number of different medical, psychological and social modules delivered by interdisciplinary teams [28,32,33,34,35,41,48,49,50]. The psychotherapy contents are mainly delivered in one-on-one sessions, but they are also delivered in group or family sessions [28,32,33,34,41]. Treatment typically lasts three weeks, with approximately eight hours of treatment per day, whether in inpatient or day-hospital treatment settings [32,33,34,41,48].

One important rehabilitation module is chronic pain education [16,35,41,49], i.e., informing patients about the bio-psycho-social model of chronic pain, the possible etiological causes and factors associated with the maintenance of pain, the consequences of inactivity and the benefit of activity despite pain. Regarding pain management, various strategies are taught, such as relaxation techniques [32,33,34,41,49,50], attention defocusing techniques [33,41,49], imagery [32,41,43,49], active daily structures [28,32,33,34,35,41,48,49,50], stress management [32,33,45,49] and problem-solving activities [32,43,49]. Physical therapy [28,32,33,34,35,41,48,49,50], biofeedback [32,33,45,49] and therapy for psychological comorbidities [33,41] such as depression or anxiety are also included in rehabilitation. In family sessions, parents actively participate in the treatment [28,32,33,34,35,41,48,49]; they are informed about how to support their children or adolescents in active pain management and how to avoid reinforcing inappropriate pain behavior. In addition, familial stress factors can be identified and worked on with the parents. Some programs also incorporate parent-only sessions [28,33,35,48]. Medical interventions include providing medical examinations [32,34,41,49] and tapering off or changing medications [28,33,41,49,50] if necessary and appropriate. Further treatment components incorporated by some of the programs address occupational therapy [28,32,33,34,35,41,49], recreational therapy [16,33,49], acupuncture/acupressure [34,49,50], diet [33,34], sleep hygiene [33,44,49], music therapy [28,41,43] and art therapy [16,28,41]. In some programs, the patients also attend some type of hospital school program [32,34,47]. To consolidate treatment strategies, the treatment plans incorporate therapeutic homework and practice [32,35,47]. Relapse prevention is an important part of therapy [32,34,41,49]. This stage is composed of stress tests, reintegration into the patient’s home and school routine, and arrangement of outpatient psychotherapy.

Some of the rehabilitation programs offer follow-up care, in which the patient’s status and goal attainment are evaluated and treatment is resumed if necessary [24,49,51]. Table 2 provides an overview of the different rehabilitation programs and their specific components. Only components reported by two or more programs in the literature are included.

### 3.3. Outcomes of Specialized Rehabilitation Programs

A recent systematic review of rehabilitation programs integrated the results of ten studies regarding the short-term effectiveness two to six months after treatment [36]. We complement these results with more recent studies and with long-term outcomes. The results are presented according to the core outcome domains from the PedIMMPACT recommendations [46].

- **Pain intensity.** The systematic review showed large short-term reductions in pain intensity [36]. More recent studies confirm these short-term reductions in pain intensity [28,34,43]. Several studies also provide evidence for long-term reductions (12 to 24–42 months after treatment) of pain intensity [16,28,34,47,52,53].
- **Satisfaction with treatment, symptoms and adverse events.** None of the studies investigated satisfaction with treatment or treatment-emergent symptoms and adverse events as outcome measures.
- **Physical functioning.** There was a large effect for the reduction of pain-related disability described in the systematic review [36]. Significant short-term effects were also found in more recent studies [28,43]. Studies also reported positive long-term effects (12 months after treatment) of rehabilitation programs on pain-related disability [28,47,52,53].
- **Emotional functioning.** The systematic review revealed a moderate effect for reduction in general anxiety, a large effect for reduction in pain-specific fear and a small to moderate effect for the reduction in depressive symptoms [36]. Benore et al. [43] replicated the short-term effects of specialized rehabilitation programs on general anxiety and on pain-specific anxiety. Sherry et al. [28] found positive short-term effects on emotional functioning that remained stable up to one year after treatment. Two more studies reported significant long-term reductions (12 months after treatment) in anxiety and depression [52,53].
- **Role functioning.** According to the systematic review, school attendance as the recommended measure of role functioning [46] was significantly improved by intensive treatment with moderate to large effect sizes [36]. Recent studies replicated these short-term effects [28,34,43]. Several studies further supported the long-term effectiveness (12 to 24–42 months after treatment) of intensive pain treatment on reducing school absence [28,34,47,52,53].
- **Sleep.** Only three studies used sleep as an outcome measure. All reported improvements in sleep disturbances [44,49,50]. One study further found stable short-term improvements in sleep onset delay, sleep duration, night waking and daytime sleepiness and an overall reduction in the use of sleep medication [44]. However, these results were based on self-report and not on validated objective measures such as actigraphy or sleep recording [46].
- **Economic factors.** A small number of studies investigated the economic effects of intensive pain treatment in terms of health care utilization and indirect costs. Significant reductions were found for health care utilization, in both the short and long term (12 to 24–42 month after treatment) [16,34,48,52]. Indirect costs, such as lost work days [16,34,52] and parental subjective financial burden [52], also showed significant reductions. Evans et al. [16] concluded that chronic pain rehabilitation is a cost-effective treatment for pediatric chronic pain.
- Regarding **moderators of treatment outcome**, the results indicated that sex [38,47], fear of pain [51], pretreatment functional impairment [53], psychological comorbidities [43,53], sleep habits [44] and patients’ readiness to self-manage pain [37] were associated with treatment outcomes. Poorer treatment outcomes were associated with female sex [38,47], high levels of fear of pain [51], low levels of school absence before treatment [53] and high levels of anxiety and depression [53]. Furthermore, decreases in anxiety [43], increases in readiness to self-manage pain [37] and improvements in sleep habits, such as sleep duration, night-waking or sleep onset, showed associations with better treatment outcomes [44].

## 4. Discussion

In this review, we aimed to provide a summary of the published specialized rehabilitation programs around the world regarding their admission criteria, treatment components and outcomes. Specialized rehabilitation programs for chronic pain in children and adolescents seem to have certain similarities around the world and have proven to be effective in treating disabling chronic pain disorders. There are many similarities regarding the admission criteria for specialized rehabilitation programs, of which pain-related disability and patient and parent motivation seem to be the most important. The different programs around the world also showed substantial similarities in structure, therapeutic orientation and individual components. All programs consist of an interdisciplinary team and include operant and cognitive-behavioral therapy, physical therapy and an active daily structure. Most of the programs address medications, use relaxation techniques and occupation therapy and have family sessions as an integral component of the treatment. The results for most of the outcome domains are comparable across all studies and indicate high short- and long-term effectiveness of specialized rehabilitation programs in pain intensity and physical, school and emotional functioning. There are, however, outcome domains, such as sleep and economic factors, for which the conclusions are rather preliminary. None of the studies investigated satisfaction with treatment or symptoms and adverse events.

### 4.1. Indications for Specialized Rehabilitation Programs

Three rehabilitation programs did not provide admission criteria. The other rehabilitation programs differed mainly in their exclusion criteria. There are large differences regarding medical issues, for example, whether patients with an underlying or active malignant disease are excluded. Furthermore, some programs include children and adolescents with psychiatric comorbidities, while others do not. This difference accounts for certain differences in treatment components. Programs that include children and adolescents with comorbidities need to be more intensive, especially concerning psychological interventions and the need to treat these comorbidities. These different exclusion criteria may further impede the comparability of the rehabilitation program outcomes, since psychological comorbidities are a risk factor for treatment failure [53]. Programs that exclude patients with comorbidities may achieve larger effects in pain-related outcome domains. Therefore, it is important that all specialized rehabilitation programs report their admission criteria to ensure that the outcomes can be interpreted accordingly.

### 4.2. Treatment Components of Specialized Rehabilitation Programs

Despite the considerable similarities between the program components, it is difficult to compare the rehabilitation programs due to the way they are described in the literature. Some programs are described in abundant detail, while others lack basic information. The descriptions of many programs are not comprehensive. Therefore, the overview of structural and therapeutic components in this review may not be considered absolute, and more similarities or even further components could arise from a more detailed and standardized description of the programs. The medical components, for example, may be considered obvious by most authors, which may be one reason why not all programs reported ongoing medical examinations. In addition, physicians are responsible for a large part of the education process to reduce the somatic fixation of the patients, and although this was not reported, it is likely an important module of most rehabilitation programs. A standardized method of reporting treatments and treatment components is desirable for investigating the similarities and differences between programs. We recommend including a detailed description in at least one publication per rehabilitation program of the characteristics outlined in Table 2, i.e., the structure, psychotherapy approach and the medical, psychological, social and other interventions. For the medical, psychological, social and other interventions, components that are essential and usually implemented should be reported. 

In addition to the differences in the components, differences between the programs could arise from the interdisciplinary team (e.g., composition, professional qualifications) or from the standardization of therapy. This information is essential and needs to be reported because a description of the components alone may not capture the core aspects of treatment. Although the rehabilitation programs can be disassembled into separate components, these components are not meant to be simply checked off to create an effective treatment. The effectiveness may instead arise from non-linear effects resulting from the strong interactions within the interdisciplinary team and from interactions between the team, the parents and the patients. These mechanisms need to be studied to precisely understand the drivers of program effectiveness to improve and strengthen existing pain centers and establish new ones.

### 4.3. Outcomes of Specialized Rehabilitation Programs

Though the short-term effectiveness of specialized rehabilitation programs has largely been demonstrated, reliable evidence regarding the long-term effectiveness over several years is lacking. One problem that arises in longitudinal clinical studies of complex interventions involves the inclusion of an appropriate control group. It is not ethically acceptable to deny severely disabled pediatric patients an effective treatment for such a long time or to provide a treatment that is clearly less effective. Randomized controlled trials (RCTs) have been conducted on psychological interventions, i.e., a subset of components of the rehabilitation programs [54,55], and one RCT has even been conducted on an entire program using a waiting-list control design [52]. However, this design only allows for short-term conclusions regarding efficacy. These RCTs have certainly contributed to good progress in the field, but there remains a need for more and stronger evidence, especially concerning long-term outcomes. One Dutch study conducted a ten-year follow-up of young adults who had received inpatient rehabilitation for chronic pain or fatigue at one of five rehabilitation centers and found that the majority of these former pediatric patients had a paid job and a moderate to good health-related quality of life [56]. However, their quality of life was somewhat lower than that of the normal population. Further long-term studies similar to the Dutch analysis are needed. Additionally, reliable evidence regarding the moderators of treatment is also needed. The existing results should be interpreted with caution due to the comparatively small sample sizes. Hirschfeld et al. [12] showed, that in regression analyses, reliable results can only be achieved with samples of several hundred patients. Such large samples require an extremely long recruitment period or a multicenter data collection process. Collaborative multicenter data collection for complex data analyses and the comparability of effectiveness of different rehabilitation programs are important areas for future research. Multicenter studies also require that comparable treatments be used at each center. Currently, this cannot be assumed, despite a certain overlap across treatment sites. 

Regarding the comparability of effectiveness, an important issue concerns the outcome measures used to indicate effectiveness. In 2008, McGrath et al. defined the PedIMMPACT recommendations for the core outcome domains and measures for pediatric acute and chronic/recurrent pain clinical trials [46]. The aim of these recommendations was to standardize the outcome domains and measures assessed in studies to facilitate their comparability and interpretation. However, not all studies adhere to these recommendations. There are, for example, huge differences in assessing pain intensity in the studies mentioned above. Although it is one of the core outcome domains, some studies do not report pain intensity [25,49]. Furthermore, some studies report pain intensity in the present moment [28,32,33], while others report pain intensity in the last 24 h [34], the last seven days [35,47,48], or the last four weeks [24]. Thus, the time period varies greatly between studies. For chronic pain, short time periods are not appropriate because chronic pain is not necessarily persistent or present each day. Thus, the time period should be long enough to also account for pediatric migraine patients, who sometimes experience attacks only once a month. Using a measure of pain intensity of less than one month may lead to distorted results. In addition, the different time periods impede the comparability of study outcomes and thereby of the effectiveness of different rehabilitation programs. The different studies also vary greatly in terms of how many outcome domains they cover. Furthermore, there are outcome domains that have not yet been investigated, despite being recommended as core outcome domains [46], such as satisfaction with treatment and symptoms and adverse events. However, to date, no validated measures exist for these domains.

Another important issue in effectiveness studies of chronic pain patients concerns the definition of what is considered effective. Is a treatment effective if the patients show statistically significant or clinically relevant changes or if the patients return to a normal functional level after treatment? In other words, what is more important: the degree of change or the status of the patient after treatment? Most studies to date have focused on the change from pre- to post-treatment or to follow-up, but what use is a significant or clinically relevant change when the patient is still severely impaired and far from normal? We may need to rethink and redefine the criteria that have to be fulfilled for the treatment to be considered effective or a success.

### 4.4. Recommendations for Future Research and Patient Care

Several areas of research need to be emphasized according to the results of this review. First, we need to devote more effort into standardizing the reporting of rehabilitation programs and outcomes to enable global comparisons of programs and their effectiveness. This review provides a set of important criteria for reporting. Furthermore, efforts should be made to report outcomes according to the PedIMMPACT recommendations in order to increase comparability. This also requires the development of valid outcome measures for different languages. Reporting a wide range of outcomes over a long follow-up gives proper consideration to the complexity of chronic pain, e.g., in some patients, certain pain symptoms may remain while pain-related disability may be substantially reduced in the long term. Additionally, we need further progress regarding long-term effectiveness, moderators of treatment outcome and mechanisms of change. Therefore, collaborative multicenter data collection may play an important role in advancing research in this field and in improving worldwide networking. Improved collaborations between pain centers and the establishment of new centers are similarly important to overcome barriers to health care utilization. The limited availability of pediatric pain clinics and centers bears the risk of additional barriers to health care. Thus, nationwide availability of appropriate health care for children and adolescents with different chronic pain conditions is one, if not the most important, goal for all countries to address pediatric chronic pain. Recently developed internet-delivered or phone-based treatment approaches may be an alternative for patients with long travel distances [57,58]. However, these programs only consist of a few of the components of specialized rehabilitation programs such as cognitive-behavioral techniques and do not replace the complexity of intensive rehabilitation programs. Thus, the establishment of new pain centers remains essential. However, training primary care providers or nurses in psychoeducation or coping skills to initiate some form of treatment before referral to specialized rehabilitation may be a good possibility to bridge the long waiting times for treatment. Comparable evaluation research is needed for quality assurance and for further development of treatment options.

### 4.5. Limitations

The results of this review should be interpreted in light of the following limitations. This review was restricted to descriptions of the structure and outcomes of specialized pain treatment programs that have been published, i.e., the programs of specialized pain centers. However, a considerable number of specialized pain clinics do not necessarily engage in research and thus do not conduct or publish studies on their programs. There may be differences in the structure or outcomes between published and unpublished specialized treatment programs. In addition, we did not conduct a systematic literature search. However, we assume that the included articles are nearly exhaustive because our review is based on a systematic review [36].

## Figures and Tables

**Table 1:** Admission criteria for specialized rehabilitation programs.

Criteria	Specialized Rehabilitation Programs								
**Inclusion**	**AUS**	**UK**	**UK**	**GER**	**USA**	**USA**	**USA**	**USA**	**USA**
	**1**	**2**	**3**	**4**	**5**	**6**	**7**	**8**	**9**
Pain for more than 3 months		x		x			x		
High pain-related disability		x	x	x	x	x	x		
Patient and parent motivation		x	x	x		x			
Failure of outpatient treatment				x	x	x			
**Exclusion**	**1**	**2**	**3**	**4**	**5**	**6**	**7**	**8**	**9**
Psychiatric needs		x			x	x			
Further assessment required		x					x		
Medical pathology/underlying disease			x		x				
Active malignant disease			x	x					
Effective medical options			x						

Only criteria explicitly reported in the literature are included for each rehabilitation program, i.e., a missing “x” does not mean that this criterion does not apply to this rehabilitation program, but that it is not reported in the literature; 1: Melbourne, AUS [50]; 2: Bath, UK [35]; 3: Bath, UK [48]; 4: Datteln, GER [41,45,47]; 5: Boston, MA, USA [32,51]; 6: Baltimore, MD, USA [49]; 7: Rochester, MN, USA [33]; 8: Cleveland, OH, USA [34,43]; 9: Philadelphia, PA, USA [28].

**Table 2:** Structural and therapeutic components of the specialized rehabilitation programs.

Components	Specialized Rehabilitation Programs								
	AUS	UK	UK	GER	USA	USA	USA	USA	USA
**Structure**	**1**	**2**	**3**	**4**	**5**	**6**	**7**	**8**	**9**
Inpatient	x			x		x		x	x
Day-hospital		x	x		x		x	x	x
Interdisciplinary team	x	x	x	x	x	x	x	x	x
**Psychotherapy Approach**	**1**	**2**	**3**	**4**	**5**	**6**	**7**	**8**	**9**
Operant and cognitive behavioral therapy	x	x	x	x	x	x	x	x	x
Acceptance and commitment therapy			x	x	x				
**Medical Interventions**	**1**	**2**	**3**	**4**	**5**	**6**	**7**	**8**	**9**
Medication	x			x		x	x		x
Medical examination				x	x	x		x	
Physical therapy	x	x	x	x	x	x	x	x	x
Biofeedback				x	x	x	x		
**Psychological Interventions**	**1**	**2**	**3**	**4**	**5**	**6**	**7**	**8**	**9**
Education		x		x		x		x	
Relaxation techniques	x			x	x	x	x	x	
Attention defocusing				x		x	x		
Imagery				x	x	x		x	
Active daily structure	x	x	x	x	x	x	x	x	x
Stress management				x	x	x	x		
Problem-solving					x	x		x	
Addressing psychological comorbidities				x			x		
**Social Interventions**	**1**	**2**	**3**	**4**	**5**	**6**	**7**	**8**	**9**
Family sessions		x	x	x	x	x	x	x	x
Parent-only sessions		x	x		x		x		x
School reintegration	x			x	x	x		x	
Patient group sessions				x	x		x	x	x
**Other**	**1**	**2**	**3**	**4**	**5**	**6**	**7**	**8**	**9**
Occupational therapy		x		x	x	x	x	x	x
Recreational therapy						x	x	x	
Hospital school program				x	x			x	
Acupressure/acupuncture	x					x		x	
Diet							x	x	
Sleep hygiene					x	x	x		
Music therapy				x				x	x
Art therapy				x				x	x
Therapeutic homework and practicing		x		x	x				
Relapse prevention				x	x	x		x	
Follow-up care				x	x	x			

Only components explicitly reported in the literature for two or more rehabilitation programs are included in this table, i.e., a missing “x” does not mean that this component is not included in this rehabilitation program, but that it is not reported in the literature; 1: Melbourne, AUS [50]; 2: Bath, UK [35]; 3: Bath, UK [48]; 4: Datteln, GER [41,45,47]; 5: Boston, MA, USA [32,44,51]; 6: Baltimore, MD, USA [49]; 7: Rochester, MN, USA [33]; 8: Cleveland, OH, USA [16,34,43]; 9: Philadelphia, PA, USA [28].
